# Molecular Mechanisms Controlling Foxp3 Expression in Health and Autoimmunity: From Epigenetic to Post-translational Regulation

**DOI:** 10.3389/fimmu.2019.03136

**Published:** 2020-02-03

**Authors:** Alessandra Colamatteo, Fortunata Carbone, Sara Bruzzaniti, Mario Galgani, Clorinda Fusco, Giorgia Teresa Maniscalco, Francesca Di Rella, Paola de Candia, Veronica De Rosa

**Affiliations:** ^1^Treg Cell Laboratory, Dipartimento di Medicina Molecolare e Biotecnologie Mediche, Università degli Studi di Napoli “Federico II”, Naples, Italy; ^2^Laboratorio di Immunologia, Istituto per L'Endocrinologia e L'Oncologia Sperimentale, Consiglio Nazionale Delle Ricerche (IEOS-CNR), Naples, Italy; ^3^Unità di NeuroImmunologia, Fondazione Santa Lucia, Rome, Italy; ^4^Dipartimento di Biologia, Università degli Studi di Napoli “Federico II”, Naples, Italy; ^5^Dipartimento di Neurologia, Centro Regionale Sclerosi Multipla, Azienda Ospedaliera “A. Cardarelli”, Naples, Italy; ^6^Clinical and Experimental Senology, Istituto Nazionale Tumori, IRCCS, Fondazione G. Pascale, Naples, Italy; ^7^IRCCS MultiMedica, Milan, Italy

**Keywords:** Foxp3, Treg cells, epigenetic regulation, Foxp3 stability, autoimmunity

## Abstract

The discovery of the transcription factor Forkhead box-p3 (Foxp3) has shed fundamental insights into the understanding of the molecular determinants leading to generation and maintenance of T regulatory (Treg) cells, a cell population with a key immunoregulatory role. Work over the past few years has shown that fine-tuned transcriptional and epigenetic events are required to ensure stable expression of Foxp3 in Treg cells. The equilibrium between phenotypic plasticity and stability of Treg cells is controlled at the molecular level by networks of transcription factors that bind regulatory sequences, such as enhancers and promoters, to regulate Foxp3 expression. Recent reports have suggested that specific modifications of DNA and histones are required for the establishment of the chromatin structure in conventional CD4^+^ T (Tconv) cells for their future differentiation into the Treg cell lineage. In this review, we discuss the molecular events that control Foxp3 gene expression and address the associated alterations observed in human diseases. Also, we explore how Foxp3 influences the gene expression programs in Treg cells and how unique properties of Treg cell subsets are defined by other transcription factors.

## Introduction

The key evidence of Forkhead box-p3 (Foxp3) as regulatory T (Treg) cell lineage-specific transcription factor is that its gene mutations lead to autoimmune disease in both mice and humans (1, 2). Foxp3 expression is used for identification of Treg cells [as Foxp3^+^ T cells in mice (1), Foxp3^high^CD45RA^−^ or Foxp3^+^CD127^−^CD25^high^ T cells in humans (3–5)], and the study of Foxp3 modulation during immune responses is crucial to understand Treg cell homeostasis and function (6). Foxp3 transcription is induced by T cell receptor (TCR) signaling and temporally persistent TCR signals activate Foxp3 transcription in self-reactive thymocytes (7, 8). Upon its expression, an autoregulatory transcriptional circuit stabilizes Foxp3 gene expression to consolidate Treg cell differentiation and activate the suppressive function (9). However, upon T cell activation, the induction of Foxp3 could represent a negative regulator of immune response (6). While Foxp3 can drive Treg cell development and function by establishing the required cell program, gene expression analysis of Foxp3^+^ and Foxp3^−^ T cells suggests that many Treg cell-specific genes are independent of Foxp3, thus changing the paradigm of Foxp3 as the only factor required for the establishment of Treg cell phenotype (10, 11). Accordingly, Foxp3 expression alone is not sufficient to convert non-Treg into Treg cells with a classical Treg-type gene signature/function. The use of chromatin-immunoprecipitation (ChIP), combined with expression array analysis, allowed the identification of several DNA sequences that are directly bound by Foxp3, which can act as transcriptional activators of some genes and repressor of others (12, 13). However, how Foxp3 controls gene expression in relation to Treg cell function is not yet fully understood. In the breach of immunological tolerance and in autoimmunity, changes in the microenvironmental cues perturb the transcriptional and epigenetic regulation of Foxp3, resulting into an impaired Treg cell generation and suppressive function (14, 15). Several studies demonstrated that, during inflammation, Treg cells may lose their phenotypic properties and be converted into effector T cells secondarily to the alteration of Foxp3 expression and stability (16–18). Thus, understanding the regulation of the mechanisms that govern Treg cell differentiation and function and whether/how this regulation may be disrupted in human autoimmune disease are of pivotal importance.

In this review, we summarize the molecular mechanisms controlling the epigenetic, transcriptional, translational, and post-translational regulation of Foxp3 in health and autoimmunity.

## Treg Cell Fate Determination and Stability

Thymus derived Treg (tTreg) cells, which differentiate intrathymically, require high-affinity or high-avidity TCR interactions with *self*-peptides/major histocompatibility complex class II (MHC II) molecules presented by either thymic epithelial cells or dendritic cells (DCs) (1, 19–22). Treg cells can also develop extrathymically by conversion of mature CD4^+^ T conventional (Tconv) cells into peripherally induced Treg (pTreg) cells, under normal homeostatic and inflammatory conditions (23–26). It is now widely recognized that Foxp3 gene regulation is responsible for both tTreg and pTreg cell generation and required for the acquisition of immunosuppressive properties, thus representing the master regulator of Treg cell lineage commitment (1, 19, 27). These Treg cell subsets are heterogeneous in terms of development, functional activity, and phenotype, but are both essential for the maintenance of immune homeostasis (28–30).

tTreg cells develop from CD4 single-positive thymocytes (31) and their Foxp3 expression is induced upon interaction with cortical and medullary thymic cells (32, 33). TCR signal strength and its duration are crucial in determining the generation of CD4 and CD8 T cell subsets during thymic differentiation (34, 35). In particular, synergistic signals downstream the TCR together with cytokine-mediated stimulation are required for the transcription of Foxp3 gene in Treg cell precursors (27, 36, 37). Together with high-affinity TCR signals, also co-stimulation through CD28 is required for tTreg cell development, as shown by the significant reduction of these cells in CD28-deficient mice as well as in mice deficient for B7-1/B7-2, the two ligands of CD28 (38, 39). Lymphocyte-specific protein tyrosine kinase (Lck) binding to the CD28 cytosolic tail is one of the events leading to Treg cell differentiation program in thymocytes (38). Since a TCR with increased *self*-reactivity can also be expressed by a non-Treg cell, other factors are necessary to drive Treg cell lineage commitment. In particular, interleukin (IL)-2 plays a fundamental role in Treg cell homeostasis and differentiation (40), a role discovered through the use of different mutant mice. Rudensky and colleagues observed reduced number of tTreg cells in IL-2-deficient compared to wild-type mice (41). However, there are some discordant studies that found normal numbers of tTreg cells in IL-2-deficient or IL-2 receptor (R)α-deficient mice (41–44), thus indicating that IL-2 may play a role in Treg cell development but may not be strictly required. IL-15, which shares the IL2-Rβ chain with IL-2, may also be involved in tTreg cell generation but, again, conflicting studies do not confirm its absolute requirement (42, 44). Transforming growth factor-β (TGF-β) is also important for the thymic differentiation of Treg cells, with mouse conditional deletion of TGF-β receptor I (TGF-βRI) in the first week of life leading to drastically reduced Treg cell differentiation (45–47). Another study has suggested a possible anti-apoptotic role for TGF-β that would enhance tTreg cell survival and thus contribute to their stability (48).

Both overlapping and distinct signaling pathways drive the generation of pTreg cells stably expressing Foxp3 such as cytokine milieu rich in TGF-β and IL-2 during antigen presentation mediated by certain DCs subsets, antigen concentration, the dose and the duration of TCR stimulation, the costimulatory molecule CD28, and IL-2/IL-2R signaling (26, 49–51). Moreover, TGF-β drives the induction of Foxp3 in pTreg cells from both murine and human Tconv cells; Foxp3 in turn downregulates the small mother against decapentaplegic (Smad)7 protein, thus suppressing the key negative regulator of TGF-β signaling (52).

Treg cell lineage is stable with minimal capacity to de-differentiate and convert into effector T (Teff) cells. Nonetheless, there exist pathological conditions in which CD25^low^ Treg cells with an unstable Foxp3 expression are converted into Tconv cells (16). Using whole-genome methylated DNA immunoprecipitation sequencing, Ohkura and colleagues have observed that Treg cells support a distinct DNA methylation pattern compared to Tconv cells and specific epigenetic mechanisms critically influence Foxp3 stability (53, 54).

Since the maintenance of a stable and functional pool of Treg cells is crucial to ensure proper immune tolerance and homeostasis, it is relevant to deeply understand the epigenetic mechanisms and factors that stabilize Foxp3, on which the balance between tolerance and autoimmunity depends.

## Epigenetic Profile of the Foxp3 Locus

**Figure 1:**
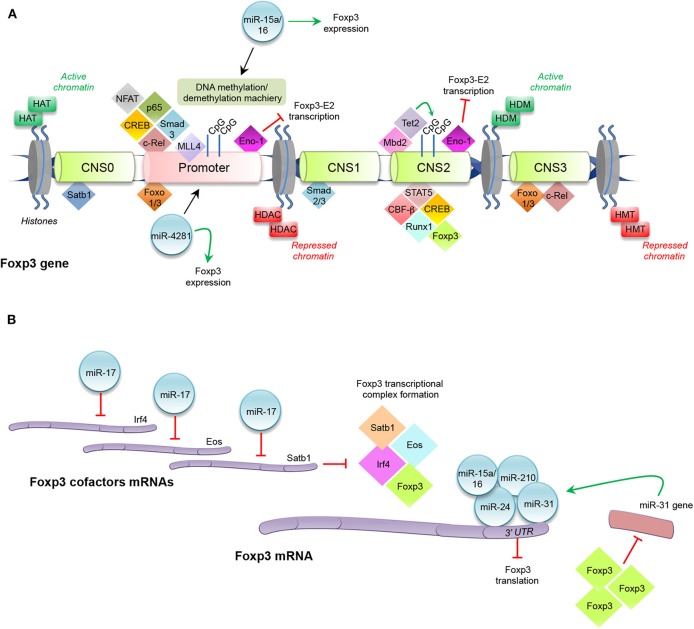
Histone modifications, transcription factors, and miRNAs regulating Foxp3 expression. **(A)** Enzymes catalyzing histone modifications: histone acetyltransferase (HAT), histone deacetylases (HDAC), histone methyltransferase (HMT), and histone demethylase (HDM). Transcription factors binding promoter and conserved non-coding sequences (CNS0, CNS1, CNS2, and CNS3) at Foxp3 locus. Promoter: nuclear factor of activated T-cells (NFAT), p65, cAMP response element binding protein (CREB), mothers against decapentaplegic (Smad)3, c-Rel, mixed lineage leukemia (MLL)4, Enolase (Eno)-1, Forkhead box-p3-Exon2 (Foxp3-E2), miR-15a/16, and miR-4281. CNS0: special AT-rich sequence binding protein (Satb)1. CNS1: Smad2 and Smad3. CNS2: Foxp3, signal transducer and activator of transcription (STAT)5, runt-related transcription factor (Runx)1-core binding factor (CBF)-β, CREB, the methyl-CpG-binding domain (Mbd)2, the chromatin-remodeling complex tet methylcytosine dioxygenase (Tet)2, Eno-1, and Foxp3-E2. CNS3: Forkhead transcription factor of the O class (Foxo)1, Foxo3, and c-Rel. **(B)** Schematic representation of miRNAs modulating Foxp3 expression: miR-17, miR15a/16, miR-210, miR-24, and miR-31. Interferon regulatory factor (Irf)4.

Increased evidence has recognized that epigenetic modifications occurring in the regulatory regions of Foxp3 locus are key determinants in Treg cell commitment (55–57). Besides the Foxp3 promoter, the three conserved non-coding sequences (CNS) within the locus, i.e., CNS1, CNS2, and CNS3, are also targets of several modifying enzymes and are regulated at different stages of Treg cell development (58). CNS1, situated downstream of the Foxp3 promoter, seems not to be essential for tTreg cell development, but reduced frequency of pTreg cells in gut-associated lymphoid tissue (GALT) and mesenteric lymph nodes (MLN) in Foxp3^ΔCNS1−gfp^ (CNS1-KO) mice instead indicates the importance of CNS1 region during Foxp3 induction in peripheral CD4^+^ T cells (58). Since GALT and maternal placenta are highly enriched in pTreg cells, CNS1-deleted mice are also characterized by increased mucosal Th2 inflammation and abortion rate (59–61). To further confirm the role of CNS1 in pTreg cell generation, Schuster and colleagues have observed that CNS1 deficiency impairs pTreg cell formation in non-obese diabetic (NOD) mice, and this phenomenon is correlated with more severe insulitis (62). DNA methylation experiments have revealed that both Foxp3 promoter and CNS2 are highly CpG demethylated in tTreg cells, opening the Foxp3 locus in Treg cell precursors and favoring Foxp3 mRNA transcription and lineage stability [Figure 1; (56, 58)]. The CNS2 region is highly rich in CpG motifs and indispensable for Treg cell lineage commitment (63–65). Demethylation of CpG motifs at the Foxp3 locus is correlated with stable Foxp3 expression in both human and mouse *ex vivo-*isolated Treg cells, while the same region is less demethylated in *in vitro*-induced Treg (iTreg) cells showing unstable Foxp3 expression (63, 66, 67). Moreover, it has been observed that IL-2-dependent stabilization of Foxp3 expression upon antigen stimulation significantly associates with demethylation of specific sequences at Foxp3 locus (68).

In the first stage of tTreg cell development, all double-positive thymocytes are highly methylated in the CNS2 region, and this epigenetic signature is also maintained in CD4^+^ single-positive Foxp3^−^ cells; a partial demethylation of CNS2 is observed in CD4^+^ single-positive Foxp3^+^ cells, and it becomes complete in mature Treg cells (69). Moreover, tTreg cells, which express Foxp3 notwithstanding CpG methylation, are not stable and lose their regulatory phenotype in the periphery (53). Toker and colleagues demonstrated that CNS2 demethylation, which begins in the early stage of tTreg cell development, occurs upon TCR stimulation and the thymic microenvironment is sufficient to enforce a regulatory identity (69). The methyl-CpG-binding domain (Mbd)2 protein binds to CNS2 and recruits both histone-modifying and chromatin-remodeling complexes, in particular tet methylcytosine dioxygenase (Tet)2, directly involved in CNS2 demethylation [Figure 1; (69–71)]. Both *in vitro* and *in vivo* murine Mbd2 deletion leads to a dramatic impairment of Treg cell suppressive function, due to a complete methylation of the CNS2 region (70). Nair and colleagues showed that in IL-2^−/−^ early developing Treg cells, Tet2 downregulation is coupled with CNS2 region methylation. Culture of IL-2-deficient tTreg cells in the presence of recombinant (r)IL-2 and observation of rIL-2-dependent Tet2 expression strongly suggest a direct role of IL-2 in Tet2 maintenance (71). CNS3-deleted CD4^+^ T cells are also unable to properly induce Foxp3, due to an impaired accumulation of mono-methylation of histone H3 at the Foxp3 promoter. Notably, Feng and colleagues observed that the impairment in Foxp3 induction is more evident in CNS3-deleted cells that received a weaker compared to cells that received a stronger TCR stimulation, thus indicating that increased TCR stimulation may partly compensate for the absence of CNS3 for the induction of Foxp3 expression (72). Recently, Kitagawa and colleagues have uncovered another regulatory CNS region, the CNS0, now considered a “super-enhancer” for Foxp3 induction in double positive thymocytes (73). CNS0 is bound by the special AT-rich sequence binding protein (Satb)1, a transcription factor that functions as a chromatin organizer, whose expression precedes Foxp3 protein appearance in Treg cell precursors, and whose deletion reduces Foxp3 expression and tTreg cell development [Figure 1; (73)]. Thus, Satb1 may be considered a “pioneer factor” during tTreg cell differentiation.

Modification of histones related to Foxp3 gene, such as histone H3 or H4 acetylation and mono-, di-, and tri-methylation of histone H3 at lysine (Lys) 4 (H3K4) or Lys 27 (H3K27), is also essential in Treg cell differentiation (74–76). Different families of enzymes catalyze these fundamental processes, which allow chromatin opening and transcriptional factor recruitment. In particular, the most important family of histone-modifying enzymes is composed of histone acetyltransferase (HAT), histone deacetylases (HDAC), histone methyltransferase (HMT), and histone demethylase (HDM) (Figure 1). These enzymes modify the N-terminal lysine or arginine residues: HAT and HDAC transfer or remove, respectively, acetyl groups to lysine residues; HMT and HDM transfer or remove one, two, or three methyl groups to/from lysine and arginine residues, respectively (77). *In vivo* HDAC3 deletion in mouse Treg cells causes lethal autoimmunity, due to an upregulation of several inflammatory-related genes, revealing HDAC3 role in promoting Treg cell development and functional activity (78). It has been reported that the methylation of H3K4 is catalyzed by a specific family of HMT, the mixed lineage leukemia (MLL) family (79). In particular, MLL4 binds to the Foxp3 promoter and 3′ untranslated region (UTR) and regulates epigenetic changes in H3K4, such as monomethylation of H3K4 (H3K4me1) (80, 81). Deletion of the MLL4-binding site by CRISPR-Cas9 technology in mice results in a decrease of Foxp3 induction in naïve CD4^+^ cells during their development, with an increase of CD4^+^CD25^+^Foxp3^−^ cells, demonstrating MLL4 requirement for the establishment of Foxp3 chromatin structure in Treg cell precursors (80).

The described finely tuned epigenetic regulation at Foxp3 locus (achieved by both DNA methylation and histone modifications) paves the way to a specific transcriptional program enforcing Foxp3 stable expression and the regulatory phenotype in Treg cells (56).

## Transcriptional Regulation of Foxp3

Several transcription factors bind either to the Foxp3 promoter or to the CNS regions to induce or maintain Foxp3 expression in tTreg cells [Figure 1; (56, 58)]. They are expressed early during Treg cell development upon TCR engagement and cytokine stimulation (i.e., IL-2, IL-15) and then bind specific DNA regions before Foxp3 protein expression (27, 36, 56). Forkhead transcription factor of the O class (Foxo)1 and Foxo3 proteins are two key regulatory determinants that induce Foxp3 expression by binding the promoter, CNS1, and CNS3 regions [Figure 1; (82–84)]. Foxo1 and Foxo3 function is tightly controlled through subcellular compartmentalization: conditions that promote Foxo nuclear localization are associated with Treg cell commitment, whereas after antigen or cytokine stimulation, these factors can be deactivated by phosphatidylinositol-3-kinase (PI3K)–Akt pathway phosphorylation that promotes their translocation from the nucleus into the cytoplasm, inhibiting the binding to Foxp3 regulatory regions (84–86). c-Rel, a member of the nuclear factor-κB (NF-κB) transcription factor family, is another important molecule, involved in Foxp3 control in tTreg cells; c-Rel deficient mice (Rel^−/−^) show reduced levels of Helios^+^Foxp3^+^ Treg cells in the periphery, due to a defective thymic development, demonstrating that c-Rel is necessary for Foxp3 expression and tTreg cell generation (87). Mechanistically, c-Rel promotes Foxp3 expression through the formation of an enhanceosome, which encompasses the transcription factors c-Rel itself, p65, nuclear factor of activated T-cells (NFAT), Smad, and cAMP response element binding protein (CREB) and induces the epigenetic changes at the Foxp3 locus, by recruiting the HAT p300 and CREB-binding protein (CBP) (88). c-Rel may also play a key role in pTreg cell generation through the binding of specific sites present in the CNS3 enhancer [Figure 1; (58, 89)]. Instead, Smad2 and Smad3 are essential for TGF-β-mediated induction of Foxp3 in pTreg cells through the binding to the intronic enhancer CNS1, important for Treg cell peripheral induction [Figure 1; (90)]. Recruitment of regulatory factors to Foxp3 locus also influences the expression of its splicing variants; in particular, it has been shown that the glycolytic enzyme enolase (Eno)-1 has an inhibitory effect on the transcription of the Foxp3 containing the exon 2 sequence (Foxp3-E2) through the binding to the promoter or to the CNS2 region of Foxp3 [Figure 1; (91)].

In tTreg cells, the complete demethylation of CpG islands is associated with the recruitment of several transcription factors, including signal transducer and activator of transcription (STAT)5, the runt-related transcription factor (Runx)1-core binding factor (CBF)-β, CREB, and Foxp3 itself [Figure 1; (92, 93)]. pTreg cells show a different signature compared to tTreg cells in terms of epigenetic modifications of Foxp3 and interaction with transcriptional factors that keep its expression stable (58, 66). Conflicting results are present in the literature concerning pTreg cell methylation pattern: some authors report demethylation of TSDR (63, 94); instead, others have shown its methylated status in pTreg cells (95).

In all, these findings concur to demonstrate that Treg cells express a unique epigenome and phenotype both in the thymus and in the periphery, finely regulated by specific enzymes and transcription factors.

## MicroRNA-Mediated Post-Transcriptional Regulation of Foxp3

MicroRNAs (miRNAs) are small (~22 nucleotides in length) non-coding RNAs, which are part of the RNA interference silencing complex (RISC), and pair to complementary sequences usually present in the 3′ UTR of mRNAs, causing mRNA decay and block of protein translation, thus influencing physiological and pathological processes (96, 97). Mice in which miRNA maturation pathway is blocked suffer from a lymphoproliferative phenotype resembling the one observed in the absence of Foxp3 itself, indicating that Treg cell development necessitates the action of miRNAs (98–100). Of all miRNAs, deletion of miR-146a-5p results in a breakdown of immune tolerance and the development of a fatal spontaneous autoimmune disorder; highlighting it is the key positive regulator of Treg cell function (101, 102). On the other hand, Foxp3 positively regulates miR-155-5p and then the coordinated action of Foxp3 and miR-155-5p blocks key inducers of the effector lineage commitment, such as Satb1 and Zinc Finger E-Box Binding Homeobox (ZEB)2 (103–106). In other words, Foxp3 imposes a multi-layered suppression of specific genes in Treg cell by both direct binding to genetic regulatory elements and by induction of miRNAs that specifically target the 3′ UTR of the same genes. Several miRNAs have been hypothesized to directly target Foxp3 3′ UTR, thus decreasing its expression level and undermining Treg cell phenotype: it is the case of miR-31, miR-24, and miR-210 [Figure 1; (107, 108)]. In particular, modulation of miR-31 in Treg cells showed a significant regulation of Foxp3, and a luciferase reporter assay suggested that the miR-31 target sequence present in the 3′ UTR of Foxp3 may indeed make Foxp3 mRNA a direct target of miR-31 action [Figure 1; (107)]. Notably, in murine Treg cells, a Foxp3 ChIP assay reported significant recruitment of the transcriptional factor to the miR-31 promoter, that indeed contains a Foxp3 binding site, suggesting the existence of a tight regulatory loop between miR-31 and Foxp3 that may be crucial in regulating Treg cell homeostasis [Figure 1; (109)]. Other miRNAs can affect Treg cell physiology also by hampering the expression of proteins that co-operate with Foxp3. MiR-17, an individual mature miRNA of the miR-17-92 cluster, has the ability to directly target Eos, interferon regulatory factor (Irf)4, and Satb1, thus indirectly reducing Foxp3 transcriptional activity [Figure 1; (110)]. A very intriguing case is that of miR-15a and miR-16 (miR-15a/16). Their expression was found decreased in umbilical cord blood Treg cells and, while the overexpression of these miRNAs leads to a reversal of Treg suppressive activity, the knockdown induces regulatory functions. A luciferase assay suggests that miR-15a/16 expression may modulate Treg function through the specific molecular binding to Foxp3 mRNA (Figure 1). In addition, miR-15a/16 overexpression is also able to reverse the demethylation profile of the Foxp3 locus, known to discriminate Treg cells from activated Foxp3^+^ Tconv cells (111), unveiling a novel mechanism by which miR-15a/16 regulate Foxp3 expression through the modulation of the DNA methylation/demethylation machinery [Figure 1; (111)].

Some miRNAs have the ability to associate with RNA polymerase II and TATA box-binding protein to bind the TATA box motifs of crucial genes, such as IL-2, insulin, and c-myc, and enhance their promoter activities and transcription initiation rate (112). MiR-4281, a miRNA specifically expressed in hominids, and the Foxp3 core promoter region share a remarkable complementary match, and the binding of the miRNA to the Foxp3 core promoter was computationally predicted and then experimentally validated: Treg cells induced in the presence of TGF-β, IL-2, and miR-4281 mimicking molecule were more stable and functional than those induced by TGF-β and IL-2 alone [Figure 1; (113)].

## Post-Translational Modification Networks regulating Foxp3

**Figure 2:**
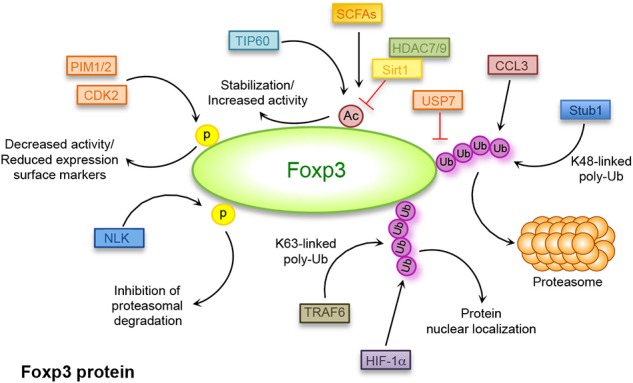
Post-translational modifications regulating Foxp3 expression. Covalent post-translational modifications in Foxp3 amino acids: ubiquitination (Ub) mediated by deubiquitinase (DUB) ubiquitin-specific-processing protease (USP)7, STIP1 homology and U-Box containing protein (Stub)1, TNF receptor associated factor (TRAF)6, chemokine (C-C motif) ligand (CCL)3, and hypoxia-inducible factor (HIF)-1α; acetylation (Ac)/deacetylation mediated by Histone deacetylase (HDAC)7, HDAC9, tat-interactive protein 60 kDa (TIP60), sirtuin (Sirt)1, and short-chain fatty acids (SCFAs); phosphorylation (p) mediated by cyclin-dependent kinase (CDK)2, Nemo-like kinase (NLK), proto-oncogene serine/threonine-protein kinase (PIM)1, and PIM2.

Regulation of Foxp3 expression and function also acts at the protein level through covalent post-translational modifications, such as ubiquitination, acetylation, and phosphorylation of different amino acids. These processes influence Foxp3 subcellular localization, functional activity, and interaction with other proteins, mainly transcriptional activators or repressors. Protein ubiquitination is a process mediated by the concerted action of a large family of ligases (E1, E2, and E3) that catalyze addition of ubiquitin peptides to lysine residues of a target protein regulating different processes, such as protein cellular trafficking or their degradation by the 26S proteasome [Figure 2; (114)]. High expression of deubiquitinase (DUB) ubiquitin-specific-processing protease (USP)7 is found in Treg cells and associates with the presence of Foxp3 in the nucleus. Treatment of cells with USP7 inhibitor results in reduced Foxp3 levels while ectopic expression of USP7 decreases Foxp3 ubiquitination and correlates with an increase of Foxp3 expression (114, 115). These findings suggest that Treg cell function can be regulated through a finely controlled mechanism consisting in the modulation of Foxp3 lysine residue ubiquitination. For example, during infection, a proper cellular response against foreign pathogens requires rapid downregulation of Treg cell number and function and the ubiquitination of Foxp3 is a signal for a rapid Treg cell switch-off (116). Moreover, in inflammatory conditions, the E3 ubiquitin ligase Stub1, expressed in response to danger signals, interacts with Foxp3 and, together with the chaperone Hsp70, catalyzes the K48-linked ubiquitination of Foxp3, leading to its downregulation [Figure 2; (116)]. The role of Stub1 in the regulation of Foxp3 level is reinforced by evidence that overexpression of Stub1 inhibits Treg cell suppressive activity and promotes a switch toward a T helper (Th)1-like phenotype, while, on the other hand, Stub1 knockdown is correlated with the inhibition of Foxp3 degradation (116).

A correlation between infection and increased ubiquitination of Foxp3 has also been reported during autoimmune diseases such as psoriasis (117). Defect in Treg cell function was already observed in psoriasis and several evidence suggested a strong correlation between infection and disease triggering (117), although a direct link had not yet been identified. Chen et al. reported that one of the links between infection and reduced Foxp3 stability in psoriasis is represented by the chemokine (C-C motif) ligand (CCL)3, which is strongly induced during infections including the one from streptococcus (117). Patients suffering from psoriasis are characterized by high CCL3 serum concentration that strongly correlates with increased degradation of Foxp3 mediated by K48-linked polyubiquitination (Figure 2 and Table 1). This phenomenon is mediated by the activation of the protein kinase B (PKB)α/Akt1 pathway (117), but the precise mechanism of how CCL3 and PKB induce polyubiquitination of Foxp3 in psoriasis is still under investigation.

**Table 1:** Foxp3 post-translational modifications (PTM) and mutations accounting for the loss of Treg cell phenotype/function and associated diseases.

**Alterations**	**Foxp3** **PTM alterations/mutation domains**	**Effect**	**Treg cell-associated defect**	**Disease**	**References**
Increased serum levels of CCL3	K48-linked polyubiquitination	Foxp3 degradation	Reduced Treg cell function	Psoriasis	(117)
Increased Sirt1 deacetylation	Reduced acetylation	Reduced Foxp3 expression	Impaired Treg cell number and suppressive function	Abdominal aortic aneurysm, Hashimoto's Thyroiditis, Grave's disease	(118–120)
Reduced TIP60 expression	Reduced acetylation	Reduced Foxp3 expression	Imbalanced Th17/Treg cell differentiation; impaired Treg cell proliferation	Rheumatoid arthritis	(121)
Germline mutations	FKH (A384T in Exon 11)	Altered interaction with TIP60	Impaired Treg cell suppressive function; impaired Foxp3 function; reduced Foxp3 stability	IPEX	(122–124)
Germline mutations	PRR (227delT, 303_304delTT in Exon 2)	Altered interaction with RORα and RORγt	Impaired Foxp3 function	IPEX	(125, 126)
Germline mutations	LZ (748_750delAAG, 750_752delGGA in Exon 7)	Impaired Foxp3 dimerization	Impaired Foxp3 function	IPEX	(127)

*Chemokine (C-C motif) ligand (CCL)3, sirtuin (Sirt)1, tat-interactive protein 60 kDa (TIP60), forkhead domain (FKH), prolin-rich region (PRR), leucine zipper (LZ), RAR-related orphan receptor (ROR)α, immune dysregulation, polyendocrinopathy, enteropathy, X-linked (IPEX)*.

Foxp3 polyubiquitination also plays a role in the regulation of Th17/Treg cell lineage fate. In hypoxic conditions, the hypoxia-inducible factor (HIF)-1, a metabolic sensor mediating the switch from oxidative metabolism to aerobic glycolysis (128), regulates the balance between Th17/Treg cells. HIF-1 promotes Th17 and inhibits Treg cell development through the binding to Foxp3 that induces its ubiquitination and subsequent degradation in the proteasome [Figure 2; (129)]. Addition of ubiquitin residues to a protein target is a signal not only associated to its degradation but to several biological processes (130). The K63-linked polyubiquitination, for example, is involved in the regulation of protein trafficking, signal transduction and protein–protein interactions (130, 131). Furthermore, Foxp3 nuclear localization, required for a proper Treg cell suppressive function, is supported by K63-linked polyubiquitination of Foxp3 mediated by the E3 ligase TNF receptor associated factor (TRAF)6 that interacts with Foxp3 catalyzing ubiquitination of the Lys 262 [Figure 2; (132)]. Also, it has been shown that in the absence of TRAF6, Treg cells display an altered suppressive function and the expression of a mutant form of Foxp3 resistant to K63 ubiquitination, unable to properly localize into the nucleus (132).

The activity of the Foxp3 transcription factor can also be regulated by other post-translational modifications, which include acetylation and deacetylation of specific lysine residues. This regulation occurs through the interaction of Foxp3 with lysine acetyl transferases (KATs also known as HATs) and lysine deacetylases (KDACs also known as HDACs) such as tat-interactive protein 60 kDa (TIP60), HDAC7, and HDAC9 [Figure 2; (133)]. Mass spectrometry analysis and structure-guided mutagenesis highlighted the presence of different acetylation sites in Foxp3 such as K31, K263, K268 (134), K250, and K252 (135). Acetylation of specific Foxp3 lysine residues augments Foxp3 stability and its ability to bind DNA (136) and activate specific effector functions [Figure 2; (134)]. This process competes with ubiquitination of the same sites and therefore increased acetylation inhibits the proteasomal degradation of Foxp3 reducing ubiquitination and *vice versa* (137).

It is well-known that sirtuin (Sirt)1-mediated deacetylation of Foxp3 associates with its reduced expression, as the result of an increased ubiquitination and subsequent degradation in the proteasome [Figure 2 and Table 1; (138)]. Treatment of cells with nicotinamide, a Sirt inhibitor, results in reduced Foxp3 degradation together with increased Treg cell number and suppressive activity (138). The function of Sirt1 is regulated by the mammalian sterile 20-like kinase (Mst)1, which increases Foxp3 acetylation and promotes its activity both indirectly, by inhibiting the activity of Sirt1, and directly, by interacting with Foxp3 and preventing its binding to Sirt1 (139). As expected, Sirt1 deletion in Treg cells correlates with higher Foxp3 expression and Treg cell function and increases allograft tolerance (140). Foxp3 acetylation also regulates the balance between Th17 and Treg cell lineage differentiation (141). A key molecule involved in this process is the transcriptional coactivator with PDZ-binding motif (TAZ) that has a pivotal role in driving Th17 cell differentiation and inhibiting Treg cell development. Indeed, TAZ is a coactivator of the Th17-specific transcriptional factor RAR-related orphan receptor (ROR)γt and constrains Treg cell differentiation by decreasing Foxp3 acetylation (141). TAZ regulates Foxp3 acetylation by competing with it for the binding to TIP60, a HAT that mediates Foxp3 acetylation and inhibits its proteasomal degradation [Figure 2; (141)].

It is important to mention that gut immune homeostasis, influenced by the composition of the commensal microbial community, is maintained, at least in part, by mechanisms controlling Foxp3 acetylation [Figure 2; (142)]. It has been shown that microbial metabolites produced by commensal bacteria, such as short-chain fatty acids (SCFAs) butyrate and propionate, promote extrathymic Treg cell generation by increasing Foxp3 acetylation (142). Impaired acetylation of Foxp3 has been associated with the pathogenesis of several autoimmune diseases, such as rheumatoid arthritis (RA) and Hashimoto's Thyroiditis (HT) (Table 1). In RA, reduced expression of Foxp3 associates with imbalanced Th17/Treg differentiation and impaired Treg cell proliferation (121). The failure of Treg cell differentiation is secondary to reduced upregulation of TIP60 acetyltransferase, which determines lower level of Foxp3 acetylation during activation of T cells from RA-affected subjects [Table 1; (121)]. A study performed in HT subjects underlined low frequency of Foxp3^+^ Treg cells, secondarily to reduced Foxp3 acetylation that leads to lower Foxp3 expression and impaired suppressive function [Table 1; (118)]. These alterations are paralleled by higher expression of the deacetylase Sirt1, suggesting a possible role of abnormal acetylation of Foxp3 in the pathogenesis of HT [Table 1; (118)]. A similar mechanism has also been described in subjects suffering from abdominal aortic aneurysm (AAA) where an autoimmune attack seems to have a pathogenic role (119). In these patients, a reduced function and percentage of Treg cells are associated with an increase of Sirt1 expression corresponding to decreased Foxp3 acetylation [Table 1; (119)]. Dysfunctions in Treg cell suppressive capacity associated with reduced acetylation of Foxp3 have also been identified in subjects with Graves' disease (GD). It has been shown that lower Foxp3 acetylation in GD is related to downregulation of miR-23a-3p that usually suppresses the expression of deacetylase Sirt1 with consequent alteration of Treg cell function [Table 1; (120)].

Foxp3 is also regulated through phosphorylation of specific sites and, depending on the phosphorylation site, it can be either activated or inhibited (143, 144). Foxp3 can be phosphorylated by the cyclin-dependent kinase (CDK)2 at four cyclin-dependent kinase (CDK) motifs (Ser/Thr-Pro) within the N-terminal repressor domain (Figure 2). These modifications are associated with reduced Treg cell suppressive ability, as CDK2-deficient Treg cells or mutations significantly increase Foxp3 and CD25 expression levels, together with a stronger Treg cell activity [Figure 2; (143)].

A novel TCR-mediated mechanism regulating Foxp3 phosphorylation involves the activation of the Nemo-like kinase (NLK), a serine/threonine protein kinase involved in regulation of cell proliferation and apoptosis (144). During T cell activation, TGF-β activates NLK that, in turn, binds and phosphorylates Foxp3 on multiple residues, inducing its deubiquitination and inhibiting the proteasomal degradation [Figure 2; (144)]. Also, under inflammatory conditions, Foxp3 is phosphorylated by the proto-oncogene serine/threonine-protein kinase (PIM)1 at the residue S422 in the C-terminal domain [Figure 2; (145)]. The pro-inflammatory cytokine IL-6 induces the expression of PIM1 that phosphorylates Foxp3, thus inhibiting Treg cell suppressive function and the expression of specific surface markers such as CD25, cytotoxic T-lymphocyte-associated protein 4 (CTLA4) and glucocorticoid-induced tumor necrosis factor receptor (TNFR)-related protein (GITR) (145). Foxp3 phosphorylation is also mediated by the kinase PIM2 that physically interacts with Foxp3 and phosphorylates multiple sites in the N-terminal domain, inhibiting Treg cell stability and suppressive function [Figure 2; (146)].

These findings indicate that regulation of Foxp3 expression and the subsequent Treg cell function rely on several molecular processes; pharmacological manipulation of these pathways may open the way for novel immunological tools to control Treg cell function and immunological tolerance in immune-related disorders.

## Induction of Treg Cell-Associated Transcriptional Programs

Foxp3 is the main defining factor of Treg cell lineage required for the induction of their functional and transcriptional program (147). However, gene-expression analysis of Foxp3^+^ and Foxp3^−^ T cells suggested that many Treg cell-specific genes are independent of Foxp3, thus changing the paradigm of Foxp3 as the only factor required for the establishment of Treg cell phenotype (11, 148). Treg cell lineage specification is indeed determined by the contribution of Foxp3-independent transcriptional programs that synergize with Foxp3 to ensure a Treg cell transcriptional signature (148). It has been observed that Treg cell fate determination is also influenced by the interplay of Foxp3 with elements related to TCR-mediated T cell activation, such as IL-2 and TGF-β signaling pathways (148, 149). Also, several Treg cell-specific molecules required for their suppressive activity—such as insulin-like 7, galectin-1, granzyme B, and Helios—are not under the transcriptional control of Foxp3 (10). In addition, Treg cell development, lineage stability, and suppressive function require CpG hypomethylation of specific sequences induced by TCR stimulation that could be fully achieved also without Foxp3 expression (53). However, although complete Treg cell differentiation requires additional transcriptional programs along with those induced by Foxp3, Foxp3 expression is crucial for survival of Treg cell precursor, Treg cell anergy, and lineage stability (28). In addition, Foxp3 induces and stabilizes the expression of genes encoding for Treg cell-specific surface markers such as CD73, CD39, TRAIL, and CTLA4 and acts as a repressor of effector cytokines induced by TCR activation such as IL-4, interferon (IFN)-γ, tumor-necrosis factor (TNF)-α, IL-17, and IL-21; this suggests that Foxp3 is necessary but not sufficient for the induction of Treg cell-specific signature (150).

The discovery of the key role of Foxp3 in the induction of Treg cell-specific transcriptional program gave rise to new questions about the mechanism of action of this transcription factor. Genome-wide analysis of Foxp3 target genes revealed that it acts both as a transcriptional activator and a repressor, through the binding to about 700 genes involved in TCR signaling pathway and maintenance of Treg cell functional programs (151, 152). Genes modulated by Foxp3 action are found both up- and downregulated in Treg cells in the thymus and in the periphery, thus changing the previous idea that Foxp3 mainly acts as a transcriptional repressor (151). However, only few Foxp3 target genes are characterized by a Foxp3 binding sequence, suggesting that the activation of Foxp3-induced transcriptional program is indirectly regulated through its cooperation with other cofactors (151). Biochemical and mass-spectrometric analyses revealed that Foxp3 might be associated with more than 360 proteins such as GATA3, NFAT, STAT3, Runx, and Foxp1, forming a large multiprotein complex of about 400–800 kDa (13). Some of these proteins are necessary for transcriptional regulation. Intriguingly, gene transcription of many Foxp3 cofactors is regulated by Foxp3 itself (13). Foxp3 is able to physically interact with sequence-specific transcription factors such as Runx1 and NFAT. The Runx1–Foxp3 complex suppresses IL-2 and IFN-γ production and induces the expression of Treg cell-specific markers such as CD25, CTLA4, and GITR (153). The key role of Foxp3 binding partners in sustaining Treg cell transcriptional program is supported by evidence showing that mutations of Foxp3 hampering its interaction with NFAT result in altered Foxp3 capacity to inhibit IL-2 secretion, upregulate CTLA4 and CD25 expression, and induce suppressor function of Treg cells (154). Moreover, the control of CTLA4 expression in Treg cells is mediated at least in part by the binding of Foxp3 to Foxp1. Indeed, a reduced binding of Foxp3 to the CTLA4 promoter region in the absence of Foxp1 has been reported (155). It has been suggested that Foxp3 interaction with NFAT prevents its binding with activator protein (AP)-1 fundamental for the induction of effector T cell responses (154). Another key partner of Foxp3 is Irf4, a transcription factor fundamental for Th2 cell differentiation. The interaction of Irf4 with Foxp3 promotes the expression of genes involved in the suppression of Th2 cell responses as suggested by the observation that Treg-specific ablation of Irf4 resulted in impaired capacity to inhibit the production of Th2 cytokines IL-4 and IL-5 (156). Foxp3 is also able to interact with GATA3, determining a reciprocal increase in their own expression; this interaction is involved in the control of Th2 responses by Treg cells (13). Foxp3 associates also with STAT3, and their interaction is lost in the presence of inhibitors of phosphorylation, suggesting that the binding is dependent on STAT3 phosphorylation state (157). The interaction between Foxp3 and STAT3 is involved in the control of Th17-mediated inflammation (13, 157). Moreover, Foxp3 is able to inhibit Th17 polarization also by directly interacting, through its exon 2 region, with the master regulator of Th17 cell lineage RORγt. The binding of Foxp3 to RORγt prevents the activation of RORγt-mediated IL-17A transcription, thus inhibiting the polarization of cells toward a Th17 phenotype (125). Hench and co-workers identified another Foxp3 binding partner, Siva; the interaction between Foxp3 and Siva potentiates the repressive effect on NF-κB activity compared to that performed by Foxp3 alone (158). Foxp3-induced activation or repression of transcription of specific target genes is also maintained through the induction of epigenetic modifications thanks to the ability of Foxp3 to bind and recruit chromatin remodeling factors. Indeed, Foxp3 can associate with TIP60 and p300 and with HDAC7 that, by adding/removing acetyl groups to/from histones, modify chromatin accessibility to other transcription factors (133). Another factor involved in Foxp3-mediated epigenetic modification is Eos, a zinc-finger transcription factor of the Ikaros family with a key role in Foxp3-dependent gene silencing and induction of Treg cell suppressive function. Foxp3 interaction with Eos is essential for the repression of IL-2 promoter in Treg cells. It has been reported that Eos binds the carboxy-terminal binding protein (CTBP)1 that recruits factors involved in histone modifications and methylation of the IL-2 promoter (159). To repress the transcription of specific target genes upregulated upon T cell activation, Foxp3 also interacts and recruits the chromatin-modifying enzyme enhancer of zeste homolog (Ezh)2 to sustain the Treg-cell specific transcriptional program during inflammatory responses (160). Ezh2, the catalytic subunit of the polycomb repressive complex (PRC)2, is involved in chromatin condensation and gene transcription inactivation by promoting the tri-methylation of Lys 27 on the N-terminal tail of the histone H3 (H3K27me3) (161). The key role of Ezh2 in the induction of Treg cell stability and function is further confirmed by the observation that Ezh2 ablation in Treg cells results in the reduction of Foxp3^+^ Treg cells in non-lymphoid tissues, inhibition of Treg cell capacity to control immune tolerance, and development of autoimmunity (162).

Although studies carried out in the last years have identified a plethora of Foxp3 cofactors, the precise mechanism by which Foxp3 regulates its target genes has not been fully understood. Kwon and co-workers proposed a model that hypothesizes the formation of two different multiprotein complexes in which Foxp3 can be alternatively integrated, located in different nuclear areas and with opposite transcriptional activity. More in detail, Foxp3 is in an active status, able to activate or repress the transcription of target genes, when part of a complex containing RelA-KAT5-IKZF2 is located at the center of the nucleus. On the contrary, when assembled in the complex containing Ezh2-IKZF3-YY1, Foxp3 is restrained at the nucleus periphery and has lower transcriptional function (163).

All these data suggest that the outcome of Treg cell transcriptional program is defined by an intricate balance between the formation of these two functional and non-functional multiprotein complexes. Moreover, Foxp3 controls the expression of several Treg cell specific genes, although several studies also highlighted the key role of some Foxp3-independent transcriptional programs in the induction of Treg cell signature.

## From naïve to Terminally Exhausted Treg Cells

It is well-known that the process of differentiation from naïve to memory cells is analogous in all T lymphocyte subsets, including Treg cells (3, 164, 165). Over the past years, the definition of naïve or memory T cell phenotype has been correlated with the expression of CD45RA or CD45RO in combination with other molecules such as CD62L, CD27, CD28, and C-C chemokine receptor 7 (CCR7) (166, 167). Accordingly, T cells may be separated in different groups: naïve T cells (CD45RA^+^CD62L^+^CCR7^+^CD27^+^CD28^+^), central-memory T cells (CD45RO^+^CD62L^+^CCR7^+^CD27^+^CD28^+^), effector-memory T cells (CD45RO^+^CCR7^−^CD62L^−^CD27^−^CD28^−^), and terminally differentiated effector T cells (CD45RA^+^CCR7^−^CD62L^−^CD27^−^CD28^−^) (167).

Several studies reported the highest frequency of naïve Treg cells in the cord blood, while they are about 6–10% of the total CD4^+^ T cells in the peripheral blood of young adults and their frequency progressively declines with age (168–172). A study by Valmori et al. showed a subset of Treg cells that are CD25^+^CCR7^+^CD62L^+^CTLA4^+^Foxp3^+^ and express the CD45RA molecule in human peripheral blood, named natural naïve Treg (NnTreg) cells. Analyses of telomere length and TCR excision circles in these cells revealed their early differentiation stage (170). In response to either TCR or autologous APC *in vitro* stimulation, NnTreg cells exhibit high proliferative capacity and partially downregulate CD45RA molecule while preserving the expression of the circulating marker CD62L. These findings suggest that NnTreg cells represent the precursors of the antigen-experienced Treg cell counterpart, which increases throughout life; moreover, the persistence of CD62L expression upon stimulation suggests that they are still able to migrate in the secondary lymphoid organs to accomplish their regulatory function (170). Accordingly, Booth et al. recently showed that memory Treg cell frequency in human subjects increases with age; however, a small fraction of naïve Treg cells expressing CD31, a marker identifying recent-migrating tTreg cells, is still observed in elderly people (>80 years) (173). These two subsets express different chemokine receptors to migrate into specific tissues. More in detail, human naïve Treg cells express high levels of the specific bone marrow homing CXC chemokine receptor type (CXCR)4, suggesting that this site represents a niche for naïve Treg cell maturation and proliferation, preceding their migration into target organs (173, 174).

Another classification of human Treg cell subpopulations has been proposed by Miyara and colleagues, on the basis of CD45RA and Foxp3 expression. More in detail, CD45RA^+^Foxp3^low^ subset identifies resting Treg (rTreg) and CD45RA^−^Foxp3^high^ activated Treg (aTreg) cells, while CD45RA^−^Foxp3^low^ represent non-Treg cells (3). In this study, the authors also revealed that rTreg and aTreg cells are both highly suppressive *in vitro*, compared to CD45RA^−^Foxp3^low^ non-Treg cells (3). Furthermore, *in vitro* and *in vivo* experiments showed that aTreg cells rise preferentially from TCR-stimulated rTreg cells, although a lower percentage may originate from CD45RA^−^Foxp3^low^ non-Treg cells (3, 175). Moreover, through a feedback mechanism, the expansion of rTreg cells is under the control of aTreg cells, and this contributes to the maintenance of their balance (3). Of note, aTreg cells express high levels of CTLA4 and display the strongest suppressive capability with terminally differentiated Treg cell features (3).

Compelling evidence reports age-associated changes in number, phenotype, and function of Treg cells. Despite the thymic involution observed in elderly subjects, Treg cell frequency increases in blood overtime, suggesting a compensatory mechanism to balance the reduced thymic function (176–179). However, prolonged antigen stimulation of Treg cells throughout life may lead to exhaustion, a process characterized by loss of effector functions (180, 181).

Exhausted T cells are usually central or effector memory characterized by the expression of specific molecules, such as programmed cell death 1 (PD-1), lymphocyte activation gene 3 (LAG-3), T cell immunoglobulin mucin 3 (TIM-3), and CTLA4. It has been described that human T cell exhaustion, resulting in loss of effector T cell function, is secondary to chronic antigenic stimulation, as those occurring during persistent infections or tumors (181). Furthermore, exhausted T cells are programmed to undergo apoptosis upon activation of the PD-1 pathway (182). Recent studies explored this end-differentiation process in depth also in Treg cells. In this context, Xiao and colleagues revealed a novel role for the molecule OX40 in the regulation of Treg cell homeostasis and function (183). OX40 is a secondary costimulatory molecule, a member of the TNFR superfamily, expressed on recently activated T cells. Several works reported that its expression and stimulation in Treg cells associate with altered suppressive ability (184–186). They showed that OX40 stimulation in Treg cells downregulates Foxp3 expression levels, leading to cell exhaustion, as confirmed by high PD-1 expression. This OX40-effect is controlled by IL-2, as exogenous addition of this cytokine could prevent Treg cell exhaustion (183). Moreover, Yang et al. suggested that liver kinase B1 (LKB1) protein, a bioenergetic sensor that controls cell metabolism and growth, is required to sustain the metabolic and immunological homeostasis of Treg cells necessary to prevent apoptosis and cell exhaustion. Indeed, loss of LKB1 in mature mice *Stk11*^fl/fl^ Treg cells upregulates PD-1 and OX40 levels, suggesting a key role for LKB1 in the prevention of exhaustion (187).

Nowadays, the differentiation process of Treg cells is not completely explored and further studies are necessary to clarify in depth the molecular pathways involved in their differentiation status and how this could be influenced by Foxp3 expression.

## Functional Plasticity and Reprogramming of Treg Cells

It is well-established that Treg cells maintain functional plasticity, modifying their transcriptional programs (34, 54). Several studies suggested that pro-inflammatory cytokines determine instability of Treg cell phenotype through the modulation of Foxp3 expression (188, 189) while others demonstrated that Foxp3 expression is particularly stable in Treg cells (94, 190). This debated issue was addressed by Hori and co-workers that proposed the “heterogeneity model” characterized by Treg cells having two different levels of commitment; on one side, there are Treg cells fully committed, resistant to conversion in other T cell subsets, while on the other, there are less committed Treg cells characterized by high degree of plasticity (191). This heterogeneity model has been demonstrated thanks to experiments showing that only a fraction of Treg cells adoptively transferred into lymphopenic mice loses Foxp3 expression and increases the production of effector cytokines becoming effector Th cells (192). Moreover, Miyara and colleagues showed that Foxp3^+^ cells are composed of CD45RA^−^Foxp3^high^ cells with suppressive capacity and non-suppressive CD45RA^−^Foxp3^low^ cells, able to secrete pro-inflammatory cytokines (3). Interestingly, the suppressive capacity of CD45RA^−^Foxp3^high^ cells correlated with the lower methylation status of the CNS2 region (13, 58, 193), important for the induction and stabilization of Foxp3 expression (58, 63, 67).

In the last few years, several reports questioned the concept that sustained expression of Foxp3 in Treg cells confers the stability of their suppressive function in different environmental conditions. Indeed, according to the transient flexibility model, Foxp3^+^ Treg cells manifest a high degree of functional plasticity and, in response to various inflammatory stimuli, can be reprogrammed into effector-like T cells, with subsequent return to their specific phenotype upon resolution of inflammation (29, 194, 195). Treg cells can also be reprogrammed in Th17 cells *via* IL-6- and IL-1β-dependent signaling. This process is mediated by activation of STAT3, RORγt, and RORα that downregulate Foxp3 expression and promote Treg cells conversion into Th17 cells (126, 196). Tsuji and colleagues have also observed that Treg cells can differentiate into follicular Th (Tfh) cells in mouse Peyer's patches by losing Foxp3 expression. The selective differentiation of Treg cells into Tfh cells guarantees the interaction of Tfh with B cells. This phenomenon has been observed only in the gut and not in other lymphoid tissues, thus indicating a specific microenvironmental cue in Peyer's patches that promotes the differentiation of Treg into Tfh cells (197).

Metabolic signals could also control Foxp3 expression and Treg cell plasticity. In particular, HIF-1α is able to control the balance between Th17- and Treg-related programs reducing Treg cell development through the induction of Foxp3 degradation (129). It has also been reported that in response to the cytokine milieu, Treg cells undergo peripheral differentiation and specialization to support the specific effector function necessary to maintain immunological homeostasis. Indeed, in the presence of IFN-γ, Treg cells promote T-bet expression that in turn induces the upregulation of CXCR3 in a mouse model of inflammation. T-bet^+^CXCR3^+^Treg cells are able to migrate into inflamed tissues and inhibit Th1 responses. On the contrary, T-bet deletion in Treg cells determines uncontrolled Th1-mediated inflammation due to an impaired migration of Treg cells into the inflammatory site (198, 199).

The complex network that regulates Treg cell plasticity, including cooperative/counteractive transcription factors, external cues, and the stable transcription of Foxp3, represents a great promise for future treatment of several immune-related diseases, including autoimmunity and cancer.

## Modulation of Foxp3 During Inflammation

**Figure 3:**
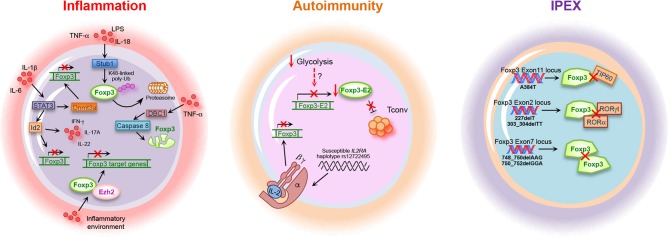
Foxp3 alterations in inflammation, autoimmunity, and IPEX. Inflammation, autoimmune diseases and IPEX (immune dysregulation, polyendocrinopathy, enteropathy, X-linked) syndrome are associated with alteration of Foxp3 gene expression, stability, and function. Lipopolysaccharides (LPS), tumor necrosis factor (TNF)-α, interleukin (IL)-18, STIP1 homology and U-Box containing protein (Stub)1, Forkhead box-p3 (Foxp3), signal transducer activator of transcription (STAT)3, Id2, and interferon (IFN)-γ, Deleted in breast cancer 1 (DBC1) factor, enhancer of zeste (Ezh)2, DNA-methyltransferase 3a (Dnmt3a), Foxp3-Exon2 (Foxp3-E2), interleukin-2 receptor alpha chain (IL-2RA), tat-interactive protein 60 kDa (TIP60), and RAR-related orphan receptor (ROR)γt.

A fine regulation of the intensity and duration of the immune response during inflammation is necessary to avoid tissue damage (200). Treg cells are crucial for maintaining immune homeostasis and prevent autoimmune diseases; on the other hand, their activity during an acute infection must be temporarily downregulated to allow adequate immune response against foreign pathogens. Moreover, increasing evidence suggests that, during inflammation, Treg cells may lose phenotypic stability and be converted into Teff cells with different phenotype (18). Several studies have been performed to understand whether inflammation drives this conversion affecting Foxp3 expression and stability. Inflammation induces post-translational modification of Foxp3, such as phosphorylation or its degradation through Stub1-mediated Foxp3 poly-ubiquitination [Figure 3; (116, 145)]. Furthermore, other mechanisms have been proposed through which inflammation regulates Foxp3 expression and stability. High amount of pro-inflammatory cytokines might favor the conversion and reprogramming of fully differentiated natural Treg (nTreg) and iTreg cells toward a Th17 phenotype during inflammation (196). In particular IL-6 has been involved in an active mechanism driving the reduction of Foxp3 expression [Figure 3; (201)]. The molecular events accounting for the conversion of Treg cells in “ex-Foxp3” Th17 cells by IL-6 involve the transcription regulator Id2, found upregulated in this cellular subset. Id2 overexpression during iTreg cell differentiation increases the expression of Th1- and Th17-related cytokines together with the reduction of Foxp3 mRNA and protein expression, suggesting a key role for this factor in the control of Treg cell stability (Figure 3). Upregulation of Id2 transcript and protein expression under inflammatory condition is promoted by the activation of STAT3, Irf4, and basic leucine zipper transcription factor, ATF-like (BATF), secondarily to the increased activity of the pro-inflammatory cytokines IL-1β and IL-6 (Figure 3). Id2 is able to inhibit the binding of the E-box binding transcription factor E2A to the Foxp3 promoter, resulting in a reduction of Foxp3 expression [Figure 3; (201)]. These findings unveil a key role for Id2 in the conversion of Treg in Th17 cells under inflammatory conditions.

Another mechanism leading to the reduction of Foxp3 expression during inflammation is mediated by the Deleted in breast cancer (DBC)1 factor, also known as p30 DBC or cell cycle and apoptosis regulator (CCAR)2 (202). Gao and co-workers recently reported that DBC1 is a Foxp3-interacting partner whose depletion in Treg cells results in the reduction of Foxp3 degradation and improvement of suppressive function in response to pro-inflammatory stimuli, such as IL-6 and TNF-α. The molecular mechanism underlying Foxp3 degradation during inflammation involves the activation of the caspase 8 degradation pathways in response to TNF-α stimulation. Indeed, treatment of cells with caspase 8 inhibitor during TNF-α treatment prevents Foxp3 degradation, thus suggesting a key role for caspase 8 in DBC1-mediated Foxp3 degradation [Figure 3; (202)].

As acute inflammation is accompanied by destruction of the surrounding host tissue, the highly tuned regulation of the duration and severity of the pro-inflammatory phase is necessary for organ/tissue regain of function (203). In this context, it has been recently shown that Treg cells can also activate specific compensatory mechanisms that augment their identity and function during inflammatory conditions (204). Indeed, the optimal expression and stability of Foxp3 require the complete demethylation of CNS2 sequence, also named TSDR, present in the first intron of the Foxp3 locus (66). Loss of Treg cell identity during inflammation has been associated with the increased expression of DNA-methyltransferase 3a (Dnmt3a)—secondarily to the activation of the IL-6-STAT3 pathway—able to methylate the CNS2, thus reducing Foxp3 expression stability [Figure 3; (65, 204)]. On the other side, prevention of Foxp3 downmodulation at the site of inflammation, such as during experimental autoimmune encephalitis (EAE), is mediated by the zinc finger protein Blimp1, which plays a key role in the control of Treg cell stability in inflamed non-lymphoid tissues by inhibiting the expression and function of Dnmt3a (204).

As previously described, the presence of pro-inflammatory cytokines is associated with downregulation of Foxp3 expression even in fully differentiated Treg cells. Moreover, it has been shown that several cytokines can also inhibit the peripheral differentiation of naïve CD4^+^ cells into Treg cells in inflamed sites. Peripheral differentiation of Treg cells has a pivotal role in the control of immune homeostasis to prevent inflammation-induced tissue damage and takes place in particular in the GALT where it is promoted by GALT DCs *via* TGF-β and retinoic acid (205, 206). It has been reported that high levels of retinoic acid could inhibit the repression of Foxp3 expression in an inflammatory microenvironment overcoming the activity of the inhibitory cytokines IFN-γ and IL-4 (207). The molecular mechanism underlying the effect of retinoic acid in enhancing Treg cell generation involves the activation of CCAAT/enhancer-binding proteins (C/EBP)—in particular C/EBPβ–conferring resistance to the effect mediated by the inhibitory cytokines (208). C/EBPβ sustains Foxp3 expression in pTreg cells by binding to the methyl-CRE sequence in the Foxp3 TSDR. During the peripheral generation of Treg cells, the Foxp3 TSDR is gradually demethylated for the induction of the phenotype. The binding of C/EBP to the still methylated Foxp3 TSDR in the early stages of the induction process could represent a defense mechanism of Foxp3 against the action of the pro-inflammatory cytokines in a phase in which its expression is still unstable. C/EBPβ-transduced Treg cells express higher level of Foxp3, display an increased suppressive function with subsequent improvement of EAE and colitis when transferred in mouse models of diseases (208). All these data suggest the presence of a mechanism mediated by retinoic acid-dependent C/EBPβ activation, which promotes and supports the peripheral induction of Treg cells in an inflammatory environment and confirm the presence of mechanisms that activate and increase Treg cell suppressive activity after an inflammatory challenge.

It has also been observed that under inflammatory conditions and during Treg cell activation, Foxp3 induces changes in chromatin accessibility modifying gene expression of activated Treg cells in response to TCR signaling and inflammatory cues. Arvey et al. reported that, although after an inflammatory stimulus Foxp3 is able to bind the same DNA regulatory elements, it boosts its transcriptional repression thanks to the binding with the chromatin modifier Ezh2 [Figure 3; (160)]. In activated Treg cells, Foxp3 associates with Ezh2, favoring its deposition at Foxp3-bound loci; this leads to inflammatory-induced transcriptional repression of specific gene and induction of suppressive activity indispensable for Treg cells to inhibit inflammation (160).

Taken together, these data suggest that, although some inflammatory cytokines are involved in Foxp3 transcriptional repression, after an inflammatory challenge, signals mediated by TCR and cytokine receptors boost Treg cell suppressive activity to inhibit inflammation. The presence of a finely and timely regulated mechanism that controls Treg cell function in the course of an inflammatory response is also confirmed by a recent study showing that inflammation- and activation-induced changes in experienced Treg cells are gradually lost overtime to avoid global immunosuppression (209).

The full comprehension of inflammation-induced mechanisms controlling Foxp3 expression and Treg cell plasticity could be useful for the identification of new molecular targets to control autoimmunity or immunodeficiency.

## Foxp3 stability and Treg Cell Function in Human Autoimmune Diseases

Defects in Foxp3 expression, Treg cell development, and function lead to several human immune diseases (14). In particular, absence or lower expression of Foxp3 protein alters suppressive function, which is tightly regulated by Foxp3 itself and by its cooperation with several cofactors (13, 151, 210). Foxp3 is a crucial regulatory transcription factor, highly necessary to induce and stabilize the specific phenotype and functional characteristics of Treg cells (56, 211). Thus, Foxp3 mediates its function through the cooperation with several transcription factors (e.g., NFAT, Runx, GATA3, and STAT3), establishing a Treg cell-specific program that can either activate or repress defined target genes (13, 151, 154, 212). For this reason, impaired Foxp3 expression leads to *in vitro* and *in vivo* defective suppressive function, resulting in T-cell mediated autoimmunity (1, 210).

The main disorder associated to Treg cell loss of function is the immune dysregulation, polyendocrinopathy, enteropathy, X-linked (IPEX) syndrome, characterized by germline mutations in the Foxp3 gene, both in non-coding and in coding sequences [Table 1; (2, 213)]. More than 60 Foxp3 point mutations have been identified so far, mainly situated in the regions encoding for the N-terminal proline-rich repressor (PRR) domain (e.g., E70H, T108M, and P187L), in the leucine zipper (LZ) domain (e.g., ΔE251), and in the DNA-binding, forkhead/winged helix (FKH) domain (e.g., R397W, I363V, and A384T) [Table 1; (122)]. All of them contribute to an impaired Treg cell development and suppressive function, leading to a systemic poly-autoimmune disease whose severity depends on the specific protein domain affected by the mutation (122). The most common IPEX mutation is the A384T, which selectively impairs Treg cell suppressive function [Figure 3 and Table 1; (122)]. Indeed, Dhuban et al. recently revealed that the A384T mutation alters the protein domain required for the interaction between Foxp3 and TIP60, necessary for its functional activation and stabilization [Figure 3 and Table 1; (123, 124)]. They showed that restoration of the Foxp3–TIP60 interaction is able to re-establish the suppressive function of Treg cells and protect mice from the development of autoimmune disorders, such as colitis or arthritis (123). Recently, several mutations in exon 2 (e.g., 227delT; 303_304delTT) and in exon 7 of Foxp3 have also been reported (e.g., 748_750delAAG; 750_752delGGA) [Figure 3 and Table 1; (127, 214–219)]. Foxp3 exon2 encodes the protein domain responsible for the binding to RORα and RORγt transcription factors (125, 126) while exon7 encodes for a sequence that is part of the LZ domain [Figure 3 and Table 1; (127)]. Thus, IPEX subjects with mutations in these exons express a functionally defective Foxp3 protein, with a consequent impaired Treg cell suppressive activity.

However, besides these genetic alterations affecting Foxp3, loss of Treg cell function in autoimmune disorders is usually unrelated to Foxp3 mutations. This is the case in multiple sclerosis (MS) and type 1 diabetes (T1D) (220). Autoimmune pathologies are characterized by the selective destruction of *self*-tissues by autoreactive T lymphocytes, due to the lack of peripheral tolerance secondary to impaired Treg cell homeostasis and/or function (14, 221, 222). Several groups showed a defective number of CD4^+^CD25^+^Foxp3^+^ Treg cells in the peripheral blood of relapsing–remitting (RR)-MS subjects (223–225). Moreover, Treg cell number inversely correlates with the expanded disability status scale (EDSS), which reflects the disability grade, thus demonstrating a strong association between Treg cell frequency and disease severity in RR-MS (127). In addition, Fletcher and colleagues found that a specific subset of Treg lymphocytes, CD39^+^Foxp3^+^, is reduced and functionally impaired in MS subjects. More in detail, they showed in healthy subjects that the Foxp3^+^ Treg cells that also express the ectonucleotidase CD39 are able to suppress IL-17 production by CD4^+^ T cells, probably removing extracellular ATP, which is necessary to promote differentiation and function of Th17 cells (226, 227). Thus, impaired expression of Foxp3 and CD39 proteins and subsequent reduced frequency and regulatory ability of this Treg cell subset could promote MS through an increase of pathogenic Th17 cell frequency (226).

Treg cell frequency has also been analyzed in brain tissue and cerebrospinal fluid (CSF) of MS subjects. Several studies showed that the absolute number of Foxp3^+^ cells is rather low or undetectable in MS brain lesions despite Treg cells being present in the CSF of MS subjects at higher frequency than in peripheral blood (228–230). However, the well-described functional impairment of peripheral Treg cells in MS subjects makes their higher frequency in CSF useless. The reduced frequency and suppressive ability described in Treg cells from RR-MS subjects are most likely due to lower Foxp3 expression, both as mRNA and protein (223, 224, 231). It is well-known that several isoforms of Foxp3 exist in human Treg cells, due to alternative splicing (127). In this context, Foxp3 splicing variants containing the sequence corresponding to the exon 2, Foxp3-E2, are considered necessary for a proper Treg cell function (127, 232, 233). Work by our group and others revealed the importance of the Foxp3-E2 in Treg cell suppressive function (91, 125, 234–236). Indeed, in healthy individuals, this subset expresses higher level of Treg cell-associated markers (e.g., CTLA4, PD-1, Ki67, and GITR) compared to the counterpart expressing the other splicing variants, confirming a major regulatory role (91, 236). We also showed that naïve-to-treatment RR-MS subjects have a reduced frequency of Foxp3-E2^+^ Treg cells (Figure 3). Moreover, the induction of Foxp3-E2^+^ iTreg from Tconv cells is also impaired due to a deranged glycolytic engagement affecting the transcriptional regulation of Foxp3-E2 (91). This defect in iTreg cell frequency and function has also been confirmed in recent-at-onset T1D subjects, demonstrating that impaired induction of Foxp3-E2 could be a common phenomenon in autoimmunity (Figure 3) (91). Accordingly, reduced function of Treg cells has also been shown in T1D individuals (237, 238). A recent study revealed that Treg cells from T1D subjects have an impaired activation, as demonstrated by the reduced frequency of the CD4^+^CD25^+^Foxp3^+^CD45RO^+^ memory Treg cell compartment (239). This impaired memory Treg cell frequency associated with reduced residual pancreatic β-cell function, evaluated as C-peptide secretion (239). Moreover, Ferraro et al. demonstrated that, although Treg cell frequency is similar to healthy controls, the expression of Foxp3 is reduced in Treg cells infiltrating the pancreatic lymph nodes (PLNs) of diabetic patients, with an increase of the Th17 counterpart. As demonstrated by the analysis of the TSDR performed in CD4^+^ T cells, the generation of “ex-Treg cells” in the PLNs of T1D patients is the result of Foxp3 protein instability (238). In all, these findings suggest impaired Treg cell activation and deranged suppressive function in T1D, as observed in MS.

Although the failure in central and peripheral tolerance secondary to reduced Treg cell frequency or function is the most common hypothesis for the development of autoimmunity, the origin of the loss of *self*-tolerance is still under debate (14, 240). Several evidence suggest that the IL-2/IL-2R pathway is necessary for Treg cell survival and functional activity, by promoting the expression of Foxp3 and other regulatory markers, such as CTLA4 (40, 41, 241–243). In this context, it has been shown that defects in IL-2/IL-2R signaling in Treg cells profoundly contribute to the development of autoimmunity since IL-2 has a key role in the control of Foxp3 expression in CD4^+^CD25^+^ cells, through the phosphorylation of STAT3 and STAT5 proteins (244–246). Moreover, deregulation of IL-2 pathway leads to altered proliferation and reduced Foxp3 expression in Treg cells from RR-MS subjects, and these parameters inversely correlate with disease clinical score (EDSS) (247). Aberrant IL-2 receptor (IL-2R) signaling has also been demonstrated in Treg cells from T1D subjects, and the T1D association in the gene region encompassing IL-2RA was firstly discovered by Vella et al. [Figure 3; (248, 249)]. More in detail, Garg and colleagues revealed that the presence of an autoimmune disease-associated IL-2RA haplotype in T1D subjects associates with reduced IL-2 responsiveness in Treg cells, which correlates with a lower Foxp3 expression and impaired suppression of CD4^+^CD25^−^ T cells [Figure 3; (250)]. In particular, it has been shown that nTreg cells isolated from T1D subjects and cultured with IL-2 show reduced Foxp3 protein stability compared to those isolated from healthy controls. This is due to a reduced IL-2 responsiveness, consequent to impaired activation of the IL-2R/STAT5 pathway (251).

Taken together, these findings underline the key relevance of Foxp3 in the control of Treg cell function and how its related defects concur to autoimmune disease pathogenesis and progression.

## Concluding Remarks

Treg cells represent a subset with a TCR bias toward *self*-epitope MHC recognition; thus, its plasticity and reprogramming into Teff cells has the potential to unleash autoimmunity. The inflammatory environment influences Foxp3 stability, in turn affecting Treg cell identity and redirecting differentiation into Teff cells. Since Treg cells acquire effector functions through the modulation of transcriptional networks controlling Foxp3 expression, its direct regulation together with the control of its cofactors represent a key immunological strategy for the treatment of autoimmune diseases. In this context, several HDAC or DNMT inhibitors (63, 252) and specific immune modulators able to neutralize proinflammatory cytokines [e.g., anti-IL2, anti-TNF-α (253, 254)] have been used to restore Foxp3 expression and Treg cell suppressive function *in vitro*, representing promising tools for future clinical trials in human autoimmune disorders. Understanding the molecular underpinnings of Foxp3^+^ Treg cell stability and the dynamics of physiological Treg cell function will shed light into their pathological dysregulation and delineate novel therapeutic strategies to halt autoimmunity.

## Author Contributions

VD conceived the work. AC, FC, SB, PC, and VD wrote the manuscript. AC, FC, SB, CF, GM, FD, and PC conceived the artwork and performed bibliographical research. MG, PC, and VD supervised the writing.

### Conflict of Interest

The authors declare that the research was conducted in the absence of any commercial or financial relationships that could be construed as a potential conflict of interest.
